# Combination Antiretroviral Therapy (cART) in Diabetes Exacerbates Diabetogenic Effects on Hippocampal Microstructure, Neurogenesis and Cytokine Perturbation in Male Sprague Dawley Rats

**DOI:** 10.3390/diagnostics12040905

**Published:** 2022-04-05

**Authors:** Jaclyn Asouzu Johnson, Robert Ndou, Ejikeme Felix Mbajiorgu

**Affiliations:** 1 School of Anatomical Sciences, University of the Witwatersrand, Johannesburg 2193, South Africa; ejikeme.mbajiorgu@wits.ac.za; 2 Department of Anatomy and Histology, School of Medicine, Sefako Makgatho Health Sciences University, Pretoria 0204, South Africa; robert.ndou@smu.ac.za

**Keywords:** diabetes, antiretroviral, cytokines, neurogenesis, hippocampus microstructure

## Abstract

The increasing incidence of diabetes and HIV/AIDS–diabetes comorbidity in society has led to the prevalence of combination antiretroviral therapy (cART) in diabetes, with some reported neural effects. Therefore, the effects of cART and type two diabetes (T2D) on the hippocampal levels of cytokines, lipid peroxidation; histomorphology and neurogenesis were investigated. Adult male Sprague–Dawley rats were divided into four groups: DB (diabetic rats); DAV (diabetic rats treated with cART (efavirenz, emtricitabine and tenofovir); AV (normal rats treated with cART) and the NC group (with no treatment). Following ninety days of treatment, the rats were terminated, and the brains excised. Immunoassay (IL-1α, IL-6, TNFα and MDA); immunohistochemical (Ki67 and DCX) and cresyl violet histomorphology analyses were carried out on brain homogenates and sections, respectively. In comparison to the control, the results showed that cART significantly elevated the IL-6, TNFα and MDA levels, while DB and DAV significantly reduced the body weight, glucose tolerance, IL-1α, IL-6, TNFα and MDA levels. The hippocampal neuronal number was reduced in AV (dentate gyrus; DG region), in the DB group (Cornu Ammonis subregion 1; CA1 and DG regions only) and in DAV (all three hippocampal regions). Additionally, the expression of neurogenic markers Ki67 and doublecortin (DCX) were reduced in the diabetic group, with a greater reduction in the cART+T2D group compared to the control. Furthermore, the neuronal number at all hippocampal regions was negatively corelated with the diabetic parameters (FBG; fasting blood glucose, NFBG; non-fasting blood glucose, AUC; area under the glucose tolerance curve) but positively correlated with body weight. Additionally, the increase in the DG neuronal nuclei area of DB and DAV was significantly positively correlated with FBG, NFBG and AUC and inversely correlated with the estimated number of neurons and neurogenesis. These findings indicate that cART in diabetes (DAV) has similar effects as diabetes relative to the induction of oxidative stress and impairment of the cytokine immune response, but exacerbated neurotoxicity is observed in DAV, as shown by a significantly decreased DCX expression compared to DB and reduction in the number of Cornu Ammonis subregion 3 (CA3) hippocampal neurons, unlike in cART or the diabetes-alone groups.

## 1. Introduction

Diabetes is a noncommunicable disease that has rapidly spread throughout South Africa and globally in recent decades [1]. Diabetes is reported to be caused by lifestyle factors [2,3], and recently, the use of cART has also been implicated as diabetogenic.

The introduction of combination antiretroviral therapy (cART) provided an effective means of suppressing human immunodeficiency virus (HIV) replication and is clinically used in Southern African regions as a first line drug for HIV and acquired immunodeficiency (AIDs) management [4]. cART consists of non-nucleoside reverse transcriptase inhibitor (NNRTI); efavirenz, a protases inhibitor (PI); emtricitabine and reverse transcriptase inhibitor (RTI) and tenofovir disoproxil fumarate. NNRTIs have been reported to induce oxidative stress [5], while PIs inhibits glucose uptake by cells, thereby stimulating an inflammatory response [6,7]. These effects of the cART component are associated with the induction of insulin resistance [8,9,10] and subsequent development of type 2 diabetes [11,12]. The diabetogenic effects of cART contributes to the fourfold increase in the prevalence of type 2 diabetes amongst HIV patients on cART compared to untreated HIV patients [13].

Type 2 diabetes (T2D) as a metabolic disorder is characterized by weight loss, hyperglycemia, insulin resistance or lack of insulin secretion in advanced stages of the disease. T2D induces oxidative stress and the perturbation of proinflammatory cytokines [14,15], which increases the incidences of infection [16] and activates a cascade of intracellular events that leads to autophagy, apoptosis and disturbances of synaptic plasticity in hippocampal neurons [17,18]. Hippocampal neurons have numerous membranes bound to glucose and insulin receptors, which are dependent on the sufficient supply of glucose for the metabolic demands of cognition and memory tasks in the hippocampus [19,20]. The potential for type 2 diabetes and components of cART to induce insulin resistance or insulin deficiency and inhibit glucose transport into neurons has been implicated in the impairment of hippocampus dependent memory functions and adult neurogenesis, especially by NNRTI in cART [21,22,23].

T2D is associated with cognitive anomalies [24] and dementia [20], while cART-induced oxidative stress has been linked to hippocampal atrophy and deficiencies in volume and synaptic plasticity [25,26], with attendant neurocognitive disorders. However, the effects of the concurrent presence of T2D and cART due to diabetes–HIV comorbidity (T2D +cART) on hippocampal histomorphology and neurogenesis remains to be documented. Meanwhile, diabetes and HIV coinfected patients abound, resulting in the prevalent use of cART amongst diabetic patients. Evidence exists for the increasing coexistence of type 2 diabetes mellitus (T2DM) among persons living with HIV/AIDs (PLWHA) [27], leading to chronic cART therapy amongst them. So far, the body of evidence has focused mostly on in vitro and MRI investigations of the independent effects of type 2 diabetes and cART on the hippocampus, and little is known about the in vivo histopathology of coexistence of the two conditions (cART+T2D) on the hippocampus relative to the induction of inflammation, perturbation of neuronal cytoarchitecture and expression of neurogenic markers. However, in HIV-positive patients, it may be difficult to ascertain if the observed oxidative stress and neuroinflammation emanates from neurotoxic interactions of cART administration in diabetes or if such effects are induced by the presence of HIV. Therefore, it is crucial to investigate and document the effects of cART administration in HIV-naïve male Sprague–Dawley diabetic rats (cART+T2D) vis-à-vis the impact on proinflammatory cytokines and lipid peroxidation levels and the likely associated changes in neuronal histomorphology and neurogenesis relative to possible cognitive and memory adverse effects.

## 2. Materials and Methods

### 2.1. Chemical and Reagents

These were obtained as follows: combination antiretroviral drug (Atripla) (Bristol-Myers Squibb and Gilead Sciences, Foster City, CA, USA), the primary antibodies (rabbit polyclonal antibody; Abcam, Cambridge, MA, USA), secondary antibody (goat anti-rabbit antibody IgGs; Vectastain, Burlingame, CA, USA) and ABC kit from (Vectastain, Burlingame, CA, USA).

### 2.2. Ethical Clearance

This study was approved by the Wits Research Animal Facility (WRAF) (approval number 2018/011/58/C) of the University of the Witwatersrand. All experimental animal handling and treatments were carried out according to the standards and principles set forth by this committee.

### 2.3. Animal Husbandry

For this study, twenty-four adult male Sprague–Dawley rats (10 weeks old) were housed separately under sterile conditions and a maintained temperature of 21–23 °C and 12-h light–dark cycle. The rats had free access to drinking water and normal rat chow for the duration of the experiment. The rats were weighed weekly, and fasting glucose tests, non-fasting glucose tests and oral glucose tolerance tests were carried out.

### 2.4. Experimental Design

Type 2 diabetes was induced in the experimental rats according to Wilson and Islam’s 2012 diabetic model through a high-fructose feed for two weeks and followed by a once-off 40-mg/kg body weight (BW) Streptozotocin (STZ) injection [28]. The screening results of random non-fasting blood glucose levels ≥250 mmol/L at the end of the third week after the onset of high-fructose feed were considered diabetic. The fasting blood glucose levels (FBG) were monitored biweekly using the Accu-Chek meter and glucose strips via tail prick after 12 h of fasting (20:00 to 08:00), while the random non-fasting blood glucose levels (NFBG) were measured and monitored biweekly using the Accu-Chek meter and glucose strips via tail prick during the evening hours without fasting to ensure that the animals remained diabetic throughout the course of the experiment. For the oral glucose tolerance test (OGTT) as an additional diagnostics of glucose tolerance for diabetes mellitus (Furman 2015), the rats were fasted for 12 h overnight (20:00 to 08:00); after which, they received an oral gavage of 2-g/kg body weight, followed by tail prick glucose monitoring using glucose strips at 0, 15, 30, 60 and 120-min intervals after glucose loading. The animals were divided into four groups of six rats each (*n* = 6). The rats in the first group were untreated and served as controls (NC), while the rats in the second group were treated with antiretroviral (cART) medication daily (consisting of efavirenz 600 mg + emtricitabine 200 mg + tenofovir 245 mg) at a directly extrapolated recommended dose of 23-mg cART/kg body weight of the animal [29] in gelatine cubes (AV). The rats in the third group (DB) were diabetic for ninety days (DB). In addition to the diabetic condition, the rats in the fourth group (DAV) received antiretroviral (cART) medication in gelatine cubes daily (DAV), as indicated above. Immediately following the confirmation of diabetes (end of third week), the cART treatment commenced, and daily treatments continued for ninety days; after which, the rats were terminated. At the end of the experiment, the rats were weighed and then anaesthetized with an overdose of sodium pentobarbitone. The hearts were perfused with 2 mL/min of 0.1-M phosphate buffer (PB) in 0.9% saline, the cranium was excised and the brain harvested and immediately separated into hemispheres. One hemisphere fixed in 0.4 M paraformaldehyde for histology and the other hemisphere stored at −80 °C for immunoassay.

### 2.5. Enzyme Linked Immunoassay of Proinflammatory Cytokines and Malondialdehyde (MDA)

Dissected brain samples from each hemisphere per animal were homogenized using handheld tissue grinder G-50 (Coyote Bioscience, Jiangusu, China) in 1:9 volume of 0.01-M phosphate-buffered saline (PBS) (pH 7.4) centrifuged at 1000× *g* for 10 min and supernatant collected. The samples were used to run an enzyme linked immunosorbent assay for Rat IL-1α, Rat IL-6, Rat TNFα and MDA. Colorimetric assay kit: TBA method (Elabscience, Houston, TX, USA; E-EL-R0011, E-EL-R0015, E-EL-R0019 and E-BC-K025-M, respectively) according to the manufacturer’s protocol. All samples were diluted 1:2, and all standards and samples were run in duplicates. Diluted samples and standards were transferred to manufacturer-coated 96-well plates and incubated for 90 min. After decanting, biotinylated detection antibody was incubated in the wells for 60 min. The wells were incubated in HRP conjugate for 30 min following aspiration and washes. Thereafter, the wells were washed and incubated in substrate reagent for 15 min and, finally, in stop solution; all incubations were at 37 °C. The optical density (OD) values were read at 450 nm. The analysis for MDA was according to the TBA colorimetric method following the manufacturer’s protocol; the concentration was calculated in μmol/g protein. The OD was read at 620 nm using an Anthos 2010 96-well plate reader coupled to ADAP software. OriginPro software was used to calculate the concentration of cytokines in the samples from the obtained OD values according to the manufacturer’s directions in pg/mL. The total protein concentration for each sample of brain homogenate was tested using Bradford reagent. The standards were made up in bovine serum albumin (BSA), and the calculated concentration of proteins in each sample was applied when calculating the final concentration of cytokines (pg/mg of protein).

### 2.6. Brian Tissue Preparation for Histology

Each brain hemisphere per animal was placed in sucrose overnight and mounted on a freezing microtome with dry ice and sucrose and cut in the sagittal plane using a frozen cryostat at 50 µm. This was repeated in one in a series six of free-floating sections collected in 0.1-M Phosphate buffer (PB) and stored in antifreeze at −20 °C until further processing. Every first series of sections was stained with cresyl violet, while the next two series of sections were used for immunohistochemical labeling of Ki67 and DCX, respectively.

### 2.7. Histology and Histomorphometry

For the cresyl violet stain, brain sections were mounted on 0.3% gelatine-coated slides and placed in 50% chloroform and 50% alcohol overnight. Six brain sections were stained with cresyl violet. These sections were serially collected 300 µm apart, starting from Bregma plate 173 to plate 177, corresponding to the rat brain atlas [30]. The sections (all groups in parallel to ensure uniform staining intensity) were then rehydrated in a degrading series of alcohol, placed in cresyl violet stain and then into distilled water. Sections were then dehydrated and mounted with a coverslip. Photomicrographs of the dorsal hippocampus of the brain sections were obtained using a Leica ICC50 HD video camera linked to a Leica DM 500 microscope (Leica Biosystems, USA). Nine camera fields from six sections per animal, from six animals per group (i.e., 36 sections per group) were captured from uniform areas within the CA1, CA3 and dentate gyrus (DG) regions of the dorsal hippocampus per group. The nuclei area and number of the neurons in the upper focal plane of these camera fields were determined using the trace and cell counter plugins of ImageJ, respectively. Only neurons with a vesicular nucleus and a centrally placed nucleoli were measured and counted. In the dentate gyrus, five counts were taken: two counts from each limb and one from the apex of the dentate gyrus. Representative photomicrographs were compiled using Microsoft Visio, and no manipulation was made to the captured images except for brightness and contrast.

### 2.8. Immunohistochemistry for Neurogenesis

From each brain hemisphere, six sections between plates 173 and 177 were selected using the atlas of a rat brain [30] for each histochemical labeling. Free-floating brain sections in 24-well plates first underwent endogenous peroxidase inhibition with 50% methanol, 50% 0.1-M PB and 1.66% hydrogen peroxide. Sections were washed, then blocked in 3% normal goat serum and 2% (BSA) and 0.25% Triton X 100 in 0.1M PB for 2 h at room temperature, followed by incubation in a polyclonal antibody raised against rabbit Ki67 and DCX (Abcam, via Biocom Africa, Pretoria, South Africa; AB15580 and AB18723, respectively) at ratios 1:1000 and 1:3000, respectively, for 48 h. The sections were washed with 0.1-M PB and incubated for 2 h with goat anti-rabbit antibody IgGs washed and incubated for 1 h with ABC solution and stained with DAB, mounted on gelatine-coated slides, dehydrated and cleared in xylene. Control sections were incubated in 0.1-M PB omitting the primary antibody. Photomicrographs of Ki67 and DCX immunolocalized dorsal hippocampus brain sections were obtained, and counts of immunolabelled cells were determined by the cell counter plugin of Image J. Only DCX immunolocalized cells in the upper focal plane with a pale centrally placed center were counted, while immunolocalized cells for Ki67 were counted in their respective clusters. Representative photomicrographs were compiled using Microsoft Visio, and no manipulation was made to the captured images except for brightness and contrast.

### 2.9. Statistical Analysis

The nuclei area and number of neurons in the hippocampal regions, concentrations of cytokines and density of immunolabeled hippocampal Ki67 and DCX were recorded as the mean ± SEM. The data were subjected to a normality test using the Shapiro–Wilks test, parametric data were analyzed using the one-way ANOVA test and a post hoc Bonferroni’s test was conducted to determine the differences between the groups. Nonparametric data were analyzed by the Kruskal–Wallis test and a post hoc Bonferroni’s pairwise analysis. The results with *p* < 0.05 were regarded as significant. All data were analyzed using IBM SPSS software, and graphs were prepared using the GraphPad prism.

## 3. Results

### 3.1. Mortality

**Figure 1:**
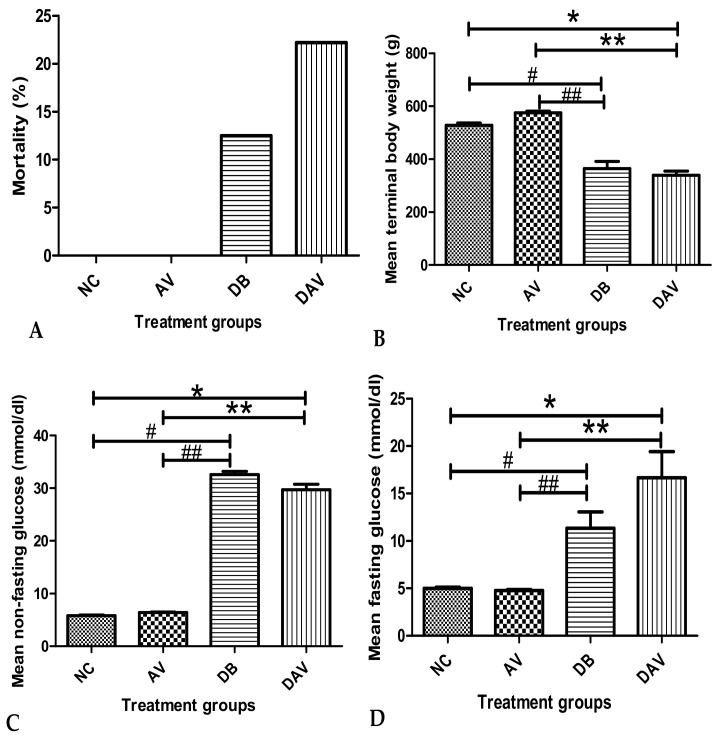
Mean mortality, terminal body weight and fasting and non-fasting blood glucose levels. (**A**) No mortality in the NC and AV groups, but (12.5%) 1 and (22.22%) 2 mortalities in DB and DAV, respectively. (**B**) Significant difference in mean body weight across groups (Kruskal–Wallis test, *p* ≤ 0.0001). ‘*’ DAV vs. NC and ‘#’ DB vs. NC (*p* = 0.034 and 0.03 respectively) and ‘**’ DAV vs. AV and ‘##’ DB vs. AV significantly decreased (*p* = 0.0001, Dunn’s pairwise post hoc test). (**C**) Significant difference in the mean NFBG between groups (Kruskal–Wallis test, *p* ≤ 0.0001), ‘*’ DAV vs. NC and ‘#’ DB vs. NC significantly increased (*p* = 0.0001 and 0.001, Dunn’s pairwise post hoc test). ‘**’ DAV vs. AV and ‘##’ DB vs. AV significantly increased (*p* = 0.05 and 0.008 respectively). (**D**) Significant difference in the mean FBG between groups (Kruskal–Wallis test, *p* ≤ 0.001). ‘*’ DAV vs. NC and ‘#’ DB vs. NC significantly increased at *p* = 0.003 and 0.008, respectively. ‘**’ DAV vs. AV and ‘##’ DB vs. AV significantly increased (*p* = 0.004 and 0.05 respectively), *n* = 6. Key: NC, Controls; AV, cART only; DB, Diabetic; DAV, diabetic with cART.

The mean percentage mortality in the DB and DAV groups was 12.5% (one out of eight animals) and 22.22% (two out of nine animals), respectively, and none in the control and AV groups. A maximum of six rats per group were randomly selected from the surviving rats in each group and used for further analysis (Figure 1A).

### 3.2. Body Weight

The mean terminal body weights in the DB and DAV groups were significantly decreased compared to the AV and NC groups. The mean body weight was highest in the AV group and lowest in the DAV group. No significant change in body weight was observed between the NC and AV and between the DB and DAV groups (Figure 1B).

### 3.3. Blood Glucose Levels

The mean NFBG levels in the DB and DAV groups were significantly increased in comparison to the levels in the NC and AV groups (Figure 1C). The mean FBG and NFBG levels for the DAV and DB groups remained significantly high throughout the experiment compared to the AV and NC groups respectively (Figure 1C,D). However, no significant difference in the mean FBG and NFBG was observed between the DB and DAV groups.

### 3.4. Oral Glucose Tolerance Test

**Figure 2:**
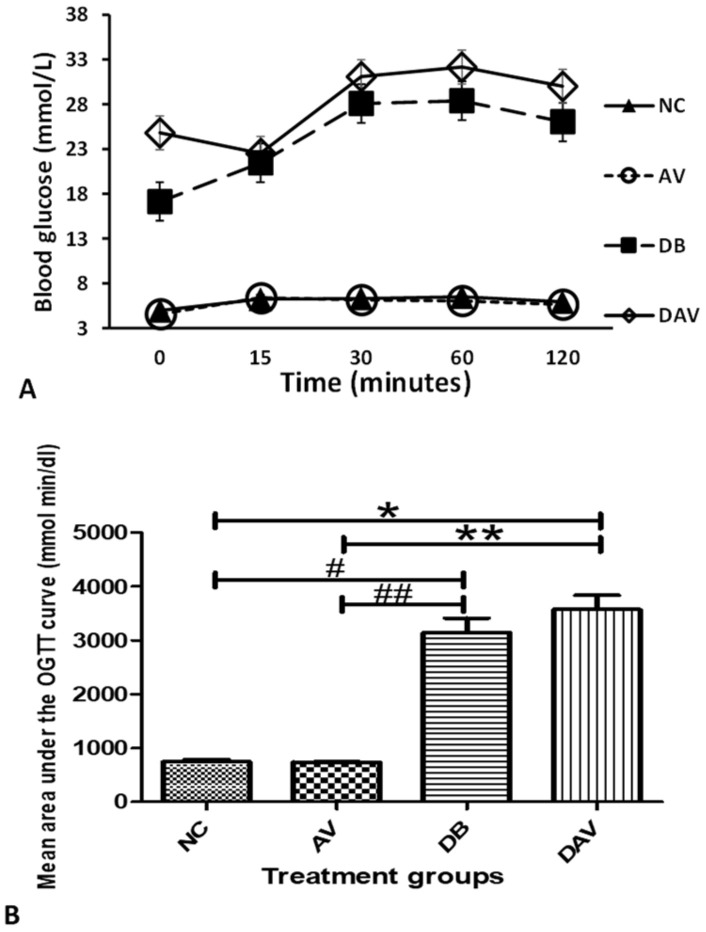
Mean oral glucose tolerance test and area under the curve. (**A**) Mean OGTT levels. (**B**) Significant difference in the mean area under the OGTT curve across groups (Kruskal–Wallis test, *p* ≤ 0.0001). ‘*’ DAV vs. NC and ‘#’ DB vs. NC significantly increased (*p* = 0.014 and 0.002) and ‘**’ DAV vs. AV and ‘##’ DB vs. AV significantly increased (*p* = 0.006 and 0.001), respectively (Dunn’s pairwise post hoc test), *n* = 6. Key: NC, Controls; AV, cART only; DB, Diabetic; DAV, diabetic with cART.

The mean oral glucose tolerance test showed optimal glucose handling in the NC and AV groups but poor glucose handling in the DB and DAV groups. After 2 h of glucose administration, the DAV had the highest level of blood glucose and area under curve (AUC), followed by the DB group; however, no difference was observed between the DB and DAV groups. The AUC was significantly increased in the DB and DAV groups compared to the NC and AV groups, and the glucose levels remained high in the DB and DAV group 2 h post-glucose load (Figure 2A,B).

### 3.5. Inflammatory Cytokines and MDA

**Figure 3:**
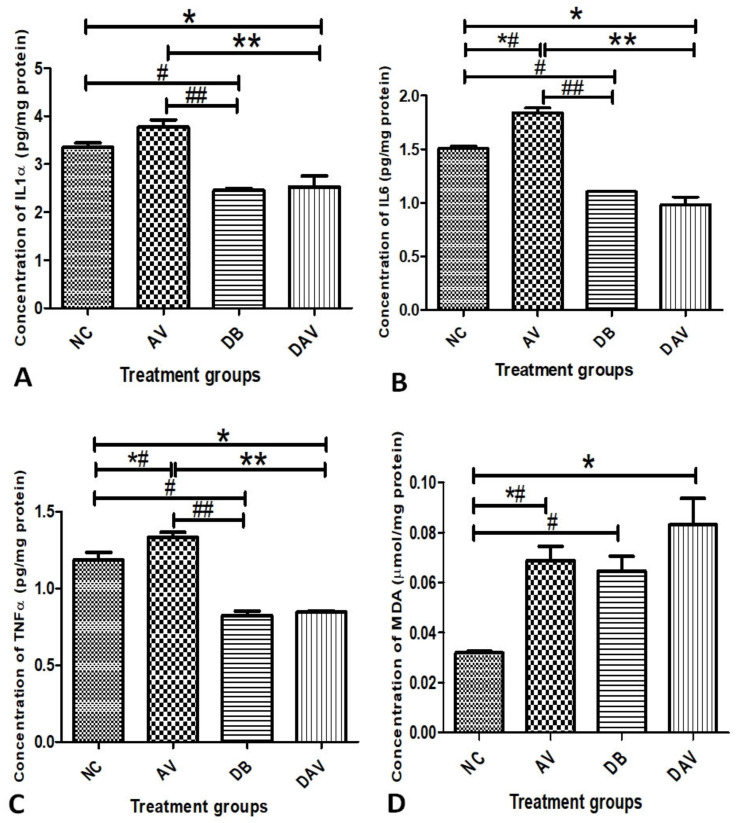
Mean terminal concentrations of IL-1α, IL-6, TNFα and MDA in hippocampal tissue homogenate. (**A**) Mean concentration of IL-1α. Significant changes in the concentration of cytokines between the groups (one-way ANOVA, *p* < 0.0001). ‘**’ DAV vs. AV and ‘##’ DB vs. AV significantly reduced (*p* = 0.0001), and ‘*’ DAV vs. NC and ‘#’ DB vs. NC also significantly reduced (*p* = 0.007 and 0.013, respectively). (**B**) Mean concentration of IL-6. Significant changes in the concentration of cytokines between the groups (one-way ANOVA, *p* < 0.0001). ‘*’ DAV vs. NC, ‘#’ DB vs. NC, ‘##’ DB vs. AV and ‘**’ DAV vs. AV significantly reduced (*p* = 0.001 and 0.0001, respectively, Bonferroni’s post hoc test). ‘*#’ AV vs. NC is significantly increased (*p* = 0.001). (**C**) Mean concentration of TNFα. Significant changes in the concentration of cytokines between the groups (one-way ANOVA *p* < 0.0001). ‘*’ DAV vs. NC, ‘#’ DB vs. NC, ‘##’ DB vs. AV and ‘**’ DAV vs. AV significantly reduced (*p* = 0.001 and 0.0001, respectively, Bonferroni’s post hoc test). ‘*#’ AV vs. NC significantly increased (*p* = 0.043). (**D**) Mean concentration of MDA in hippocampal tissue homogenate. Significant changes in the concentration of MDA between the groups (one-way ANOVA, *p* < 0.0001) ‘*’ DAV vs. NC, ‘*#’ AV vs. NC and ‘#’ DB vs. NC are significantly increased compared to the control (*p* = 0.013, 0.029 and 0.001, respectively, Bonferroni’s post hoc test), *n* = 4. Key: NC, Controls; AV, cART only; DB, Diabetic; DAV, diabetic with cART.

The concentrations of proinflammatory cytokines IL-1α, IL-6 and TNFα in the hippocampal tissue homogenate are as shown in Figure 3A–C, respectively. The concentrations of all three cytokines (IL-1α, IL-6 and TNFα) in DB and DAV were significantly reduced compared to the NC (*p* = 0.007, 0.013 and 0.0001, respectively) and AV groups (*p* = 0.001). The concentrations of all three cytokines (IL-1α, IL-6 and TNFα) in the AV groups were significantly increased compared to the control (NC) (*p* = 0.04, 0.001 and 0.043, respectively) (Figure 3A–C). Similarly, the MDA levels significantly increased in AV, DB and DAV compared to the control (NC) (*p* = 0.019, 0.039 and 0.001, respectively) (Figure 3D).

### 3.6. Histopathology of the Hippocampus

#### 3.6.1. Number of Neurons in Hippocampal Regions

**Figure 4:**
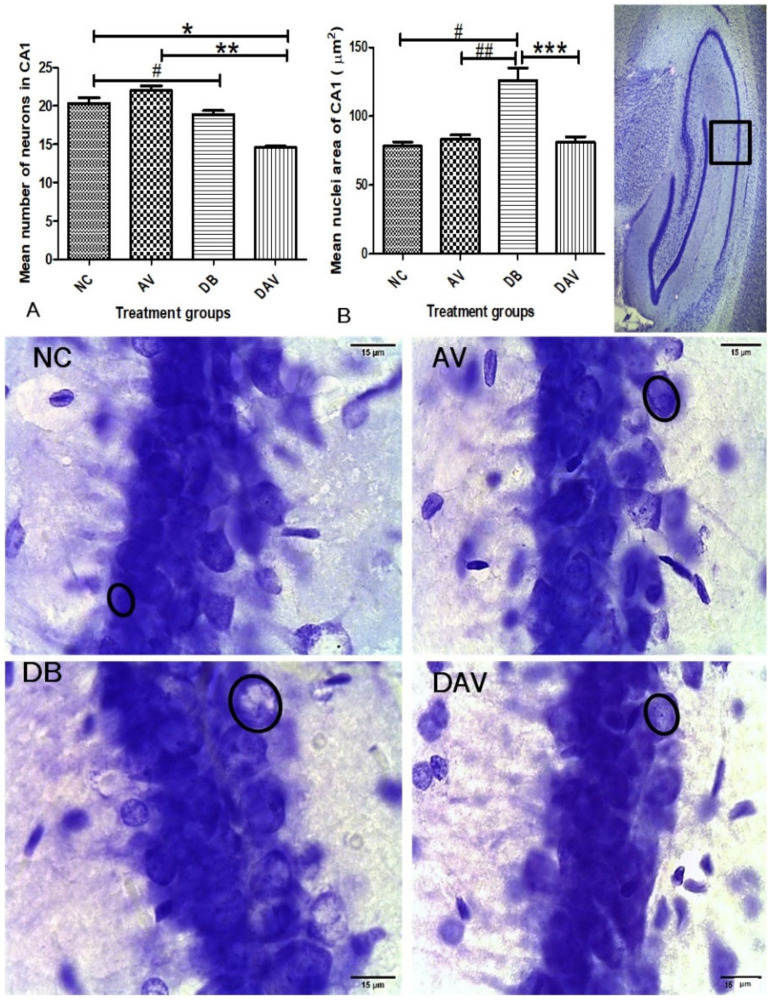
Mean nuclei area and number of neurons in the CA1 region of the dorsal hippocampus. (**A**) Significant changes in mean number of neurons of the CA1 region (Kruskal–Wallis test, *p* ≤ 0.0001) ‘*’ DAV vs. NC and ‘#’ DB vs. NC are significantly decreased (*p* = 0.037 and 0.0001, respectively). ‘**’ DAV vs. AV significantly decreased (*p* = 0.001). (**B**) Significant changes in the mean nuclei area between the groups (Kruskal–Wallis test, *p* ≤ 0.005). ‘#’ DB vs. NC, ‘##’ DB vs. AV and ‘***’ DB vs. DAV are significantly increased (*p* = 0.001,0.016 and 0.005, respectively). Circles indicate boundaries of neuronal nuclei, and squares indicate specific regions from which micrographs and measurements were made. Scale bar = 15 µm, *n* = 6. Key: NC, Controls; AV, cART only; DB, Diabetic; DAV, diabetic with cART.

**Figure 5:**
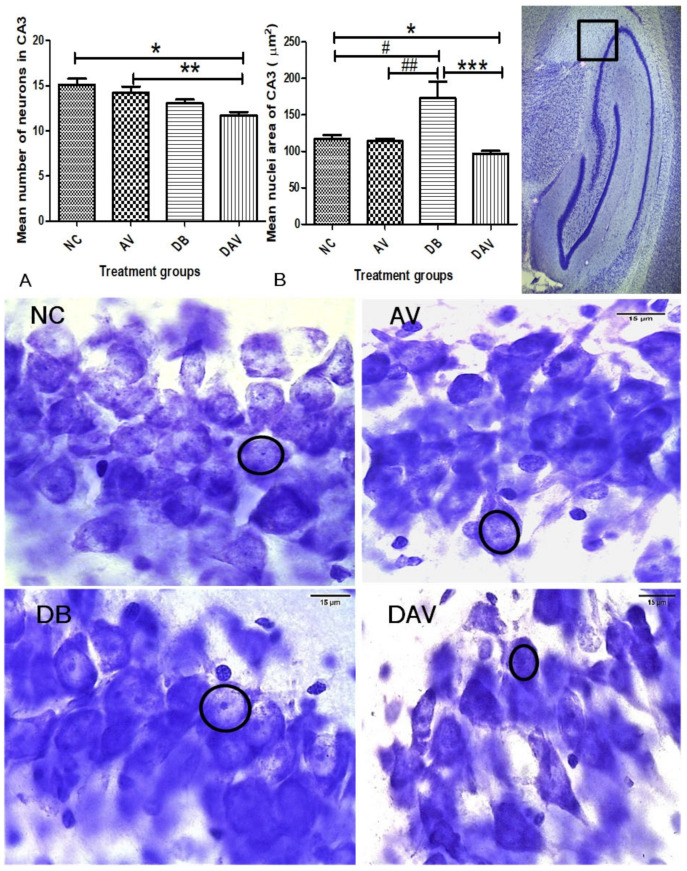
Mean nuclei area and number of neurons in the CA3 region of the dorsal hippocampus. (**A**) Significant changes in the mean number of neurons of the CA3 region (one-way ANOVA test, *p* ≤ 0.001). ‘*’ and ‘**’ are significantly decreased (*p* = 0.001 and 0.017, respectively). (**B**) Significant changes in the mean nuclei area in the CA3 region between the groups (Kruskal–Wallis test, *p* ≤ 0.005). ‘#’ DB vs. NC, ‘##’ DB vs. AV and ‘***’ DB vs. DAV are significantly increased (*p* = 0.041, 0.016 and 0.0001, respectively). ‘*’ DAV vs. NC is significantly reduced (*p* = 0.045). Circles indicates boundaries of the neuronal nuclei, and squares indicate the specific area from which micrographs were taken and measurements made. Scale bar = 15 µm, *n* = 6. Key: NC, Controls; AV, cART only; DB, Diabetic; DAV, diabetic with cART.

**Figure 6:**
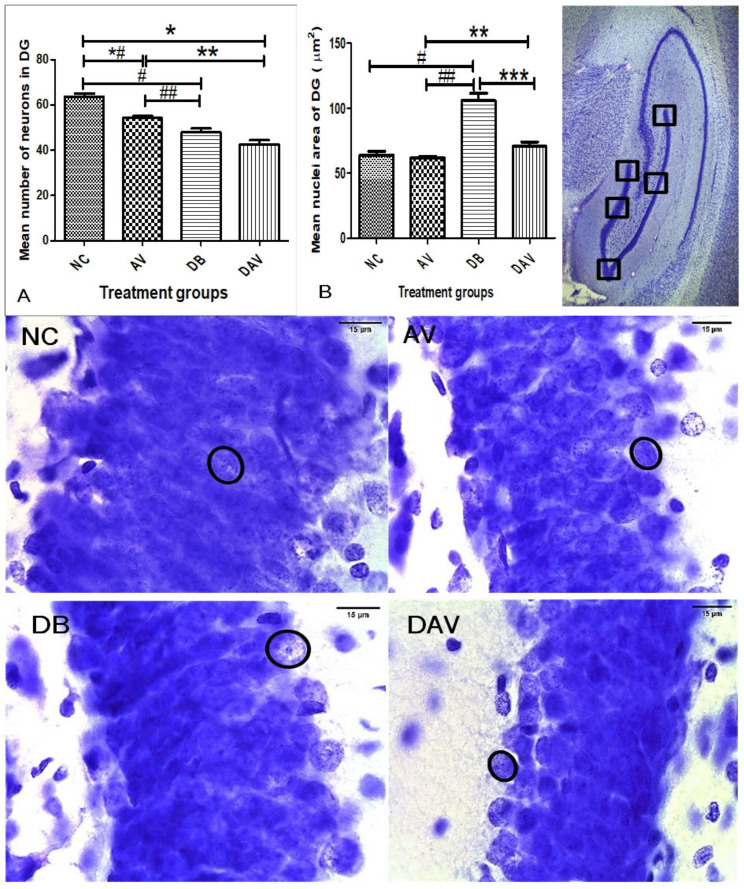
Mean nuclei area and number of neurons in the dentate gyrus region of the dorsal hippocampus. (**A**) Significant changes in the mean number of neurons in the DG region (one-way ANOVA test, *p* ≤ 0.0001). ‘*’ DAV vs. AV, ‘*#’ AV vs. NC and ‘#’ DB vs. NC are significantly decreased (*p* = 0.001, 0.0001 and 0.0001, respectively). ‘**’ DAV vs. AV and ‘##’ DB vs. AV are significantly decreased (*p* = 0.0001). (**B**) Significant changes in the mean nuclei area in the DG region between the groups (Kruskal–Wallis test, *p* ≤ 0.005). ‘#’ DB vs. NC, ‘##’ DB vs. AV and ‘***’ DB vs. DAV are significantly increased (*p* = 0.0002, 0.0001, and 0.0001, respectively). ‘**’ DAV vs. AV, ‘#’ DB vs. NC, ‘##’ DB vs. AV and ‘***’ DB vs. DAV are significantly increased (*p* = 0.045). Circles indicate boundaries of neuronal nuclei, and squares indicate specific regions from which micrographs were taken and measurements made, *n* = 6. Scale bar = 15 µm. Key: NC, Controls; AV, cART only; DB, Diabetic; DAV, diabetic with cART.

In the control groups, a wider and densely clustered row of cell can be seen observed in all hippocampal regions (Figure 4, Figure 5 and Figure 6). In the treated groups (AV, DB and DAV), gradual reduction in the neuronal population is observed, especially in the CA3 region of the DAV group, where the neurons appear scanty (Figure 5). The mean estimated number of neurons in all regions of the hippocampus of the DAV group was significantly decreased compared to the control (NC) and AV groups (Figure 4, Figure 5 and Figure 6). The mean neuronal number in the CA1 region of the DB group was significantly decreased in comparison with the mean neuronal number in the NC group (*p* = 0.037). Similarly, the mean neuronal number significantly reduced in the dentate gyrus of the DB compared to the NC and AV groups (*p* = 0.0001 and 00.034, respectively) and reduced in the AV compared to the NC group (*p* = 0.001) (Figure 6).

#### 3.6.2. Neuronal Nuclei Area in Hippocampal Regions

The nuclei area of the DB groups is observed to be consistently increased compared to other groups at all hippocampal regions (circles; Figure 4, Figure 5 and Figure 6). While the mean nuclei in the DAV are slightly increased in the CA1 and DG regions but appear apoptotic in the CA3 region, incidentally, the mean neuronal number of the DAV group is also significantly reduced in this region (circles; Figure 5). The mean nuclei area of the neurons in all three regions of the hippocampus was significantly increased (*p* ≤ 0.041, 0.016 and 0.005) in the DB group compared to the other groups (NC, AV and DAV), except comparison with the DAV in the DG region (Figure 4, Figure 5 and Figure 6). However, the mean nuclei area of neurons in the DAV significantly decreased compared to the NC group in the CA3 region (Figure 6).

### 3.7. Immunohistochemistry for Neurogenesis in the Hippocampus Double Cortin and Ki67

**Figure 7:**
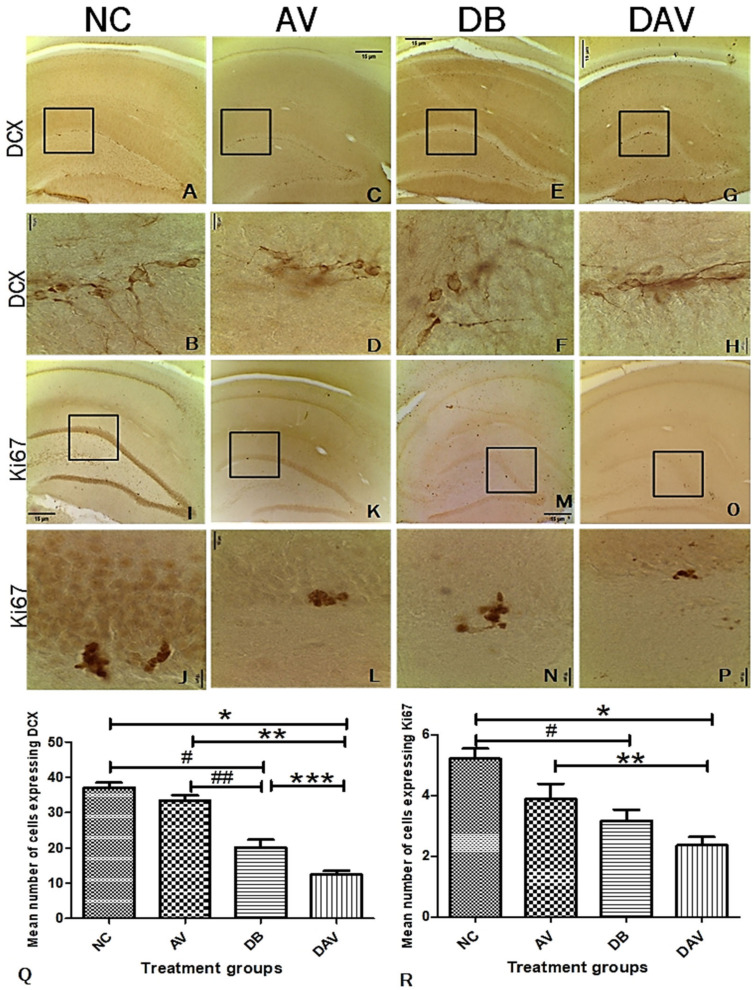
Photomicrograph of the hippocampus showing the expression of DCX in the subgranular zone of the dentate gyrus region. (**A**,**B**) Expression of DCX in the control, showing numerous clusters of cell bodies and projections expressing DCX. (**C**,**D**) Expression of DCX in the AV group: several small pockets of cells and projections expressing DCX. (**E**,**F**) Expression of DCX in the DB group: few cells and their projections expressing DCX. (**G**,**H**) Expression of DCX in the DAV group: minimal number of cells expressing DCX, most of the DCX expression were observed in the projections. (**I**–**P**) Expression of Ki67 showing gradual reduction in Ki67 expression across the groups NC, AV, DB and DAV, respectively. (**Q**) Significant difference in the expression of DCX between groups (one-way ANOVA, *p* = 0.0001). ‘*’ DAV vs. NC, ‘**’ DAV vs. AV, ‘#’ DB vs. NC and ‘##’ DB vs. AV are significantly decreased (Bonferroni’s post hoc, *p* = 0.0001). ‘***’ DAV vs. DB significantly decreased (Bonferroni’s post hoc, *p* = 0.011). (**R**) Significant difference in expression of Ki67 between the groups (one-way ANOVA, *p* = 0.0001). ‘*’ DAV vs. NC and ‘#’ DB vs. AV are significantly decreased (Bonferroni’s post hoc, *p* = 0.007 and 0.0001, respectively). ‘**’ DAV vs. AV is significantly decreased (Bonferroni’s post hoc, *p* = 0.05). Photomicrographs at ×100; squares indicate the area captured at ×1000 magnification, *n* = 6. Scale bar = 15 µm. Key: NC, Controls; AV, cART only; DB, Diabetic; DAV, diabetic with cART.

The number of neurons expressing DCX in the DB and DAV groups was significantly decreased compared to the NC and AV groups (*p* = 0.0001), and the expression of DCX in the DAV was also significantly decreased compared to DB (*p* = 0.011; Figure 7A–H,Q). Additionally, a significant reduction was observed in Ki67 expression in the DB and DAV groups compared to the NC group (*p* = 0.007 and 0.0001, respectively), and the DAV was significantly reduced compared to the AV group (*p* < 0.051; Figure 7I–P,R). There were no significant differences between the control and AV in both DCX and Ki67 expressions.

### 3.8. Correlation of Histomorphology and Neurogenic Parameters

Evaluation of the possible correlations between different parameters showed significant negative correlations between the mean body weight and diabetic parameters (FBG, NFBG and AUC). Similarly, the mean estimated number of neurons (in CA1N, CA3N and DGN) and neurogenic markers (Ki67 and DCX) significantly positively correlated with BW and negatively with FBG, NFBG and AUC (Table 1). Additionally, a significantly positive correlation was observed independently between the mean number of neurons in CA1N, CA3N and DGN (Table 1) and between neuronal nuclei area in CA1A, CA3A and DGA (Table 2). Furthermore, the DGA has a strong significant positive correlation with FBG, NFBG, AUC and the nuclear area (CA1A and CA3A) but a significantly negative relationship with BW and number of neurons in CA1N, CA3N and DGD (*p* = 0.011, 0.032 and 0.011, R = −0.509, −0.439 and −0.510, respectively) and neurogenic markers (Table 2).

## 4. Discussion

The diabetic brain is susceptible to oxidative damage, which has been linked to neuro-inflammation and autophagy [15]. Obesity, hyperglycemia and impaired glucose tolerance [31] with an attendant elevation of MDA [32] and proinflammatory cytokines [33] have been identified as risk factors in the pathogenesis of type 2 diabetes. Similar effects of significantly elevated body weight; cytokines (IL-1α, IL-6 and TNFα) and MDA levels were observed in the cART-treated animals. However, hyperglycemia and impaired glucose tolerance were not observed in the cART-treated groups (AV), as shown by the FBG, NFBG and AUC levels, suggesting the initiation but not establishment of metabolic perturbation in this group. Significantly elevated FBG, NFBG, AUC, lipid peroxidation levels and weight loss observed in the diabetic groups (DB and DAV) are consistent with a previous report of hyperglycemia, oxidative stress and lipodystrophy associated with advanced stages of T2D [34,35,36].

Cytokines in the brain may arise from circulatory invasion or produced by support cells (microglial and astrocytes) and neurons in response to neuroinflammation and neuroprotection triggers [37,38]. Additionally, diabetes is characterized as an inflammatory condition [39] with elevated levels of cytokines often reported in the serum and tissue of T2D patients and animal models [12,40]. However, inflammation, oxidative stress and hyperglycemia together induce perturbation in immunometabolism, which impairs the immune system [41]. These immune compromises shown by the decreased production of cytokines has been reported in diabetic patient immune cells [42,43]. Therefore, the significantly reduced cytokines (IL-1α, IL-6 and TNFα) levels in the hippocampus of the DB and DAV groups, contrary to previous reported increases [12,33,40], may suggest a compromised immune response and prolonged diabetes. Furthermore, cytokines are essential for long-term plasticity learning and memory functions [44], and as such, the reduction of cytokines in the DB and DAV groups compared to the control may suggest a compromise in the cognitive and memory processes of the hippocampus. Since the cytokine levels were significantly reduced in diabetes and cART-treated HIV-naïve rat groups, it is possible that diabetic–HIV coinfected patients on chronic cART treatment may be predisposed toward greater inflammatory disturbances and, therefore, require more clinical attention in their diagnosis and management. Additionally, the significantly increased MDA levels in the DB and DAV groups suggest that the cellular and functional integrity of the hippocampus will be more susceptible to neurotoxic effects due to cART treatment in diabetes.

Previous reports have indicated that cART and diabetes are both independently implicated in hippocampal neurodegeneration [45,46]. Our results collaborate these reports with a significant decrease in the number of neurons in the AV group (in DG region); DB group (in CA1 and DG regions) and in all the hippocampal regions in the DAV group (i.e., CA1, DG and CA3). Additionally, the nuclei area of the DAV is significantly reduced compared to the control. This indicates that cART treatment in diabetes exacerbated and extended hippocampal neuronal decline into the CA3 region, which is not affected by either cART or diabetes alone. The decline in DG and CA3 neurons will adversely affect the associated functions in cognition, pattern separation, episodic memory and pattern completion, as has been previously reported [47]. This is because CA3 neurons play a crucial role in memory processes, with recurrent internal and wide-ranging connectivity [48,49] to other hippocampal regions and cortices. Therefore, the reduction in CA3 neuron density and nuclei area in the DAV group may imply a reduction in the connectivity of this region within the hippocampus and may result in adverse functional defects.

The sub-granular zone of the hippocampal dentate gyrus is one of the established zones in the brain for adult hippocampal neurogenesis. The significantly decreased expression of DCX and Ki67 in the diabetic (DB) and cART-treated diabetic animal (DAV) groups infers that the proliferation of hippocampal stem cells, as well as migrating immature neurons, were significantly impaired. Such compromised stem cell generation and migration may diminish the hippocampal potential to recover from inherent apoptotic processes. Interestingly, the number of neurons at different hippocampal regions is positively significantly correlated with Ki67 and DCX expressions and negatively with FBG, NFBG and AUC, which is consistent with reports of a persistent decline in adolescent hippocampal neurogenesis due to increased cell death of immature differentiating neurons [50]. This also suggests that elevated levels of diabetes parameters may exacerbate the loss of hippocampal neurons and induce a decline in neurogenesis. Additionally, the DG nuclei area positive correlation with the diabetic parameters (FBG, NFBG and AUC) and mean nuclei areas of the CA1 and CA3 regions but inverse correlation with neuronal density (in all regions of the hippocampus) and neurogenic parameters (Ki67 and DCX) may indicate a possible central functional role associated with the DG nuclei. Furthermore, the significant increase in neuronal nuclei area and significant reduction in mean neuronal number in the DB group observed in all hippocampal regions may be due to necrosis, which is morphologically characterized by cell and nuclear swelling in the initial stages of cell death [51,52].

In summary, our results indicate that cART therapy in the diabetic condition elevates lipid peroxidation but reduces body weight; proinflammatory cytokines; number of neurons (CA1, CA3 and DG); proliferation of new cells and migration of immature neurons in the dentate gyrus with likely adverse implications in hippocampal overall function. Additionally, cART treatment in diabetic conditions may induce heightened oxidative stress and may worsen the previously reported CNS infections, memory and cognitive loss involving diabetes or cART independently. The clinical implications of these findings (though in the HIV-naïve rat model) hinges on the chronic cART regimen amongst diabetes patients with HIV infection and, therefore, calls for caution in the clinical diagnosis and management of these groups of patients.

## Figures and Tables

**Table 1:** Spearman’s rho nonparametric correlation of diabetes parameters with neuronal number and neurogenic parameters.

	Diabetes Parameters				Neuronal Number			Neurogenic Markers
	BW	FBG	NFBG	AUC	CA1N	CA3N	DGN	Ki67
**FBG**								
*p* value	0.0001 *							
*R* value	−0.613							
**NFBG**								
*p* value	0.0001 *	0.0001 *						
*R* value	−0.648	0.761						
**AUC**								
*p* value	0.0001 *	0.0001 *	0.0001 *					
*R* value	−0.723	0.729	0.691					
**CA1N**								
*p* value	0.0001 *	0.0001 *	0.0001 *	0.0001 *				
*R* value	0.716	−0.808	−0.706	−0.762				
**CA3N**								
*p* value	0.011 *	0.0001 *	0.002 *	0.004 *	0.0001 *			
*R* value	0.508	−0.821	−0.593	−0.563	0.794			
**DGN**								
*p* value	0.001 *	0.0001 *	0.0001 *	0.0001 *	0.0001 *	0.0001 *		
*R* value	0.629	−0.900	−0.800	−0.692	0.824	0.794		
**KI67**								
*p* value	0.038 *	0.0001 *	0.001 *	0.006 *	0.0001 *	0.001 *	0.0001 *	
*R* value	0.426	−0.735	−0.637	−0.546	0.715	0.639	0.758	
**DCX**								
*p* value	0.0001 *	0.0001 *	0.0001 *	0.0001 *	0.0001 *	0.0001 *	0.0001 *	0.0001 *
*R* value	0.667	−0.882	−0.714	−0.777	0.796	0.745	0.838	0.748

Key: BW; body weight, FBG; fasting blood glucose, NFBG; non-fasting blood glucose, AUC; area under the OGTT curve. Neuronal number at CA1, CA3 and DG (CA1N, CA3N and DGN, respectively. ‘*’ Significant correlation (*p* < 0.05), R value; Correlation Coefficient.

**Table 2:** Spearman’s rho nonparametric correlation of diabetes parameters with neuronal nuclei area and neurogenic parameters.

	Diabetes Parameters				Neuronal Nuclei Area			Neurogenic Markers
	BW	FBG	NFBG	AUC	CA1A	CA3A	DGA	Ki67
**CA1A**								
*p* value	0.101	0.08	0.014 *	0.245				
*R* value	−0.343	0.364	0.497	0.247				
**CA3A**								
*p* value	0.68	0.493	0.277	0.698	0.0001 *			
*R* value	−0.89	−0.147	0.231	−0.083	0.719			
**DGA**								
*p* value	0.0001 *	0.006 *	0.0001 *	0.012 *	0.0001 *	0.009 *		
*R* value	−0.717	0.546	0.706	0.505	0.696	0.52		
**KI67**								
*p* value	0.038 *	0.0001 *	0.001 *	0.006 *	0.296	0.526	0.047 *	
*R* value	0.426	−0.735	−0.637	−0.546	−0.222	0.136	−0.409	
**DCX**								
*p* value	0.0001 *	0.0001 *	0.0001 *	0.0001 *	0.19	0.208	0.035 *	0.0001 *
*R* value	0.667	−0.882	−0.714	−0.777	−0.277	0.266	−0.433	0.748

Key: BW; body weight, FBG; fasting blood glucose, NFBG; non-fasting blood glucose, AUC; area under the OGTT curve. Neuronal nuclei area at CA1, CA3 and DG (CA1A, CA3A and DGA, respectively). ‘*’ Significant correlation (*p* < 0.05), R value; Correlation Coefficient.

## Data Availability

The minimal dataset for the results from this study will be made available through a University of the Witwatersrand archived link.

## References

[1] Pheiffer C., Pillay-van Wyk V., Joubert J.D., Levitt N., Nglazi M.D., Bradshaw D. (2018). The prevalence of type 2 diabetes in South Af-rica: A systematic review protocol. BMJ Open.

[2] Al-Maskari F. (2010). Lifestyle Diseases: An Economic Burden on the Health Services. UN Chronicle Magazine UN. https://www.un.org/en/chronicle/article/lifestyle-diseases-economic-burden-health-services.

[3] Feng R., Du S., Chen Y., Zheng S., Zhang W., Na G., Li Y., Sun C. (2015). High carbohydrate intake from starchy foods is positively associated with metabolic disorders: A Cohort Study from a Chinese population. Sci. Rep..

[4] Meintjes G., Moorhouse M.A., Carmona S., Davies N., Dlamini S., Van Vuuren C., Manzini T., Mathe M., Moosa Y., Nash J. (2017). Adult antiretroviral therapy guidelines 2017. South. Afr. J. HIV Med..

[5] Fokunang C.N., Hitchcock J., Spence F., Tembe-Fokunang E.A., Burkhardt J., Levy L., George C. (2005). An Overview of Mitochondrial Toxicity of Nucleoside Reverse Transcriptase Inhibitors Associated with HIV Therapy. Int. J. Pharmacol..

[6] Osuji F.N., Onyenekwe C.C., Ahaneku J.E., Ukibe N.R. (2018). The effects of highly active antiretroviral therapy on the serum levels of pro-inflammatory and anti-inflammatory cytokines in HIV infected subjects. J. Biomed. Sci..

[7] Zhou H., Jarujaron S., Gurley E.C., Chen L., Ding H., Studer E., Pandak W.M., Hu W., Zou T., Wang J.-Y. (2007). HIV protease inhibitors increase TNF-α and IL-6 expression in macrophages: Involvement of the RNA-binding protein HuR. Atherosclerosis.

[8] Honnapurmath V.K., Patil V.W. (2017). Antiretroviral therapy-induced insulin resistance and oxidative deoxy nucleic acid damage in human immunodeficiency virus-1 patients. Indian J. Endocrinol. Metab..

[9] Noor M.A. (2007). The role of protease inhibitors in the pathogenesis of HIV-associated insulin resistance: Cellular mechanisms and clinical implications. Curr. HIV/AIDS Rep..

[10] Rehman K., Akash M.S.H. (2016). Mechanisms of inflammatory responses and development of insulin resistance: How are they interlinked?. J. Biomed. Sci..

[11] Calle M., Fernandez M. (2012). Inflammation and type 2 diabetes. Diabetes Metab..

[12] Tsalamandris S., Antonopoulos A.S., Oikonomou E., Papamikroulis G.-A., Vogiatzi G., Papaioannou S., Deftereos S., Tousoulis D. (2019). The Role of Inflammation in Diabetes: Current Concepts and Future Perspectives. Eur. Cardiol. Rev..

[13] Brown T.T., Cole S.R., Li X., Kingsley L.A., Palella F.J., Riddler S.A., Visscher B.R., Margolick J.B., Dobs A.S. (2005). Antiretroviral therapy and the prevalence and incidence of diabetes mellitus in the multicenter AIDS cohort study. Arch. Intern. Med..

[14] Berbudi A., Rahmadika N., Tjahjadi A., Ruslami R. (2020). Type 2 Diabetes and its Impact on the Immune System. Curr. Diabetes Rev..

[15] Muriach M., Flores-Bellver M., Romero F.J., Barcia J.M. (2014). Diabetes and the Brain: Oxidative Stress, Inflammation, and Autophagy. Oxidative Med. Cell. Longev..

[16] Alves C., Casqueiro J., Casqueiro J. (2012). Infections in patients with diabetes mellitus: A review of pathogenesis. Indian J. Endocrinol. Metab..

[17] Gonzalez C., Lee M.-S., Marchetti P., Pietropaolo M., Towns R., Vaccaro M., Watada H., Wiley J.W. (2011). The emerging role of autophagy in the pathophysiology of diabetes mellitus. Autophagy.

[18] Razavi S., Sadeghi A., Hami J., Esfandiary E., Hejazi Z. (2016). The effect of diabetes mellitus on apoptosis in hippocampus: Cellular and molecular aspects. Int. J. Prev. Med..

[19] McNay E.C., Ong C., McCrimmon R., Cresswell J., Bogan J., Sherwin R.S. (2010). Hippocampal memory processes are modulated by insulin and high-fat-induced insulin resistance. Neurobiol. Learn. Mem..

[20] McNay E.C., Recknagel A.K. (2011). Reprint of: ‘Brain insulin signaling: A key component of cognitive processes and a potential basis for cognitive impairment in type 2 diabetes’. Neurobiol. Learn. Mem..

[21] Jin J., Grimmig B., Izzo J., Brown L.A.M., Hudson C., Smith A.J., Tan J., Bickford P., Giunta B. (2016). HIV Non-Nucleoside Reverse Transcriptase Inhibitor Efavirenz Reduces Neural Stem Cell Proliferation in Vitro and in Vivo. Cell Transplant..

[22] Ramos-Rodriguez J.J., Molina-Gil S., Ortiz-Barajas O., Jimenez-Palomares M., Perdomo G., Cozar-Castellano I., Lechuga-Sancho A., Garcia-Alloza M. (2014). Central Proliferation and Neurogenesis Is Impaired in Type 2 Diabetes and Prediabetes Animal Models. PLoS ONE.

[23] Stranahan A.M., Arumugam T., Cutler R.G., Lee K., Egan J.M., Mattson M.P. (2008). Diabetes impairs hippocampal function through glucocorticoid-mediated effects on new and mature neurons. Nat. Neurosci..

[24] Kodl C.T., Seaquist E.R. (2008). Cognitive Dysfunction and Diabetes Mellitus. Endocr. Rev..

[25] Opii W.O., Sultana R., Abdul H.M., Ansari M.A., Nath A., Butterfield D.A. (2007). Oxidative stress and toxicity induced by the nucleoside reverse transcriptase inhibitor (NRTI)—2′,3′-dideoxycytidine (ddC): Relevance to HIV-dementia. Exp. Neurol..

[26] Robertson K., Liner J., Meeker R.B. (2012). Antiretroviral neurotoxicity. J. NeuroVirology.

[27] Kousignian I., Sautereau A., Vigouroux C., Cros A., Kretz S., Viard J.P., Slama L. (2021). Diagnosis, risk factors and management of diabetes mellitus in HIV-infected persons in France: A real-life setting study. PLoS ONE.

[28] Wilson R.D., Islam M.S. (2012). Fructose-fed streptozotocin-injected rat: An alternative model for type 2 diabetes. Pharmacol. Rep..

[29] Frampton J.E., Croom K.F. (2006). Efavirenz/Emtricitabine/Tenofovir Disoproxil Fumarate. Drugs.

[30] Paxinos G., Watson C. (2006). The Rat Brain in Stereotaxic Coordinates: Hard Cover Edition.

[31] (2007). American Diabetes Association Diagnosis and Classification of Diabetes Mellitus. Diabetes Care.

[32] Manohar S.M., Vaikasuvu S.R., Deepthi K., Sachan A., Narasimha S.R.P.V.L. (2013). An association of hyperglycemia with plasma malondialdehyde and atherogenic lipid risk factors in newly diagnosed Type 2 diabetic patients. J. Res. Med. Sci. Off. J. Isfahan Univ. Med. Sci..

[33] AlZamil H. (2020). Elevated Serum TNF-α Is Related to Obesity in Type 2 Diabetes Mellitus and Is Associated with Glycemic Control and Insulin Resistance. J. Obes..

[34] American Diabetes Association 2 (2015). Classification and Diagnosis of Diabetes. Diabetes Care.

[35] Benedé-Ubieto R., Estévez-Vázquez O., Ramadori P., Cubero F.J., Nevzorova Y.A. (2020). Guidelines and Considerations for Metabolic Tolerance Tests in Mice. Diabetes Metab. Syndr. Obes. Targets Ther..

[36] Pournaghi P., Sadrkhanlou R.-A., Hasanzadeh S., Foroughi A. (2012). An investigation on body weights, blood glucose levels and pituitary-gonadal axis hormones in diabetic and metformin-treated diabetic female rats. Vet. Res. Forum.

[37] Muhammad M. (2020). Tumor Necrosis Factor Alpha: A Major Cytokine of Brain Neuroinflammation. Cytokines.

[38] Salmeron K.E., Maniskas M.E., Edwards D.N., Wong R., Rajkovic I., Trout A., Rahman A.A., Hamilton S., Fraser J.F., Pinteaux E. (2019). Interleukin 1 alpha administration is neuroprotective and neuro-restorative following experimental ischemic stroke. J. Neuroinflamm..

[39] Donath M.Y., Shoelson S.E. (2011). Type 2 diabetes as an inflammatory disease. Nat. Rev. Immunol..

[40] Herrero L., Shapiro H., Nayer A., Lee J., Shoelson S.E. (2010). Inflammation and adipose tissue macrophages in lipodystrophic mice. Proc. Natl. Acad. Sci. USA.

[41] Daryabor G., Atashzar M.R., Kabelitz D., Meri S., Kalantar K. (2020). The Effects of Type 2 Diabetes Mellitus on Organ Metabolism and the Immune System. Front. Immunol..

[42] Martinez N., Ketheesan N., Martens G.W., West K., Lien E., Kornfeld H. (2016). Defects in early cell recruitment contribute to the increased susceptibility to respiratory Klebsiella pneumoniae infection in diabetic mice. Microbes Infect..

[43] Richard C., Wadowski M., Goruk S., Cameron L., Sharma A.M., Field C. (2017). Individuals with obesity and type 2 diabetes have additional immune dysfunction compared with obese individuals who are metabolically healthy. BMJ Open Diabetes Res. Care.

[44] Bourgognon J.-M., Cavanagh J. (2020). The role of cytokines in modulating learning and memory and brain plasticity. Brain Neurosci. Adv..

[45] Akang E.N., Dosumu O.O., Afolayan O.O., Fagoroye A.M., Osiagwu D.D., Usman I.T., Oremosu A.A., Akanmu A.S. (2019). Combination antiretroviral therapy (cART)-induced hippocampal disorders: Highlights on therapeutic potential of Naringenin and Quercetin. IBRO Rep..

[46] Madhusudhanan J., Suresh G., Devanathan V. (2020). Neurodegeneration in type 2 diabetes: Alzheimer’s as a case study. Brain Behav..

[47] Scharfman H.E., Myers C.E. (2016). Corruption of the dentate gyrus by “dominant” granule cells: Implications for dentate gyrus function in health and disease. Neurobiol. Learn. Mem..

[48] Cherubini E., Miles R.M. (2015). The CA3 region of the hippocampus: How is it? What is it for? How does it do it?. Front. Cell. Neurosci..

[49] Le Duigou C., Simonnet J., Teleñczuk M.T., Fricker D., Miles R.M. (2014). Recurrent synapses and circuits in the CA3 region of the hippocampus: An associative network. Front. Cell. Neurosci..

[50] Broadwater M.A., Liu W., Crews F.T., Spear L.P. (2014). Persistent Loss of Hippocampal Neurogenesis and Increased Cell Death following Adolescent, but Not Adult, Chronic Ethanol Exposure. Dev. Neurosci..

[51] Elmore S. (2007). Apoptosis: A review of programmed cell death. Toxicol. Pathol..

[52] Kwon H.-K., Lee J.-H., Shin H.-J., Kim J.-H., Choi S. (2015). Structural and functional analysis of cell adhesion and nuclear envelope nano-topography in cell death. Sci. Rep..

